# Computed tomography pitfalls and diagnostic value in a patient with post-infarction ventricular septal defect, cardiogenic shock, and iatrogenic type B aortic dissection during extracorporeal membrane oxygenation support: a case report

**DOI:** 10.1093/ehjcr/ytag524

**Published:** 2026-07-14

**Authors:** Adrià Ruiz-López, Mario Salido Iniesta, Sònia Gómez Revelles, Claudia Taroncher Domingo, David Viladés Medel

**Affiliations:** Cardiology Department, Hospital de la Santa Creu i Sant Pau, Universitat Autònoma de Barcelona, Carrer St. Antoni M. Claret 167, Barcelona 08025, Spain; Cardiology Department, Hospital Universitari Mútua Terrassa, University of Barcelona, Pl. Doctor Robert 5, Terrassa, Barcelona 08221, Spain; Cardiology Department, Hospital de la Santa Creu i Sant Pau, Universitat Autònoma de Barcelona, Carrer St. Antoni M. Claret 167, Barcelona 08025, Spain; Cardiology Department, Hospital Universitari Mútua Terrassa, University of Barcelona, Pl. Doctor Robert 5, Terrassa, Barcelona 08221, Spain; Cardiology Department, Hospital Universitari Mútua Terrassa, University of Barcelona, Pl. Doctor Robert 5, Terrassa, Barcelona 08221, Spain; Cardiology Department, Hospital de la Santa Creu i Sant Pau, Universitat Autònoma de Barcelona, Carrer St. Antoni M. Claret 167, Barcelona 08025, Spain; Cardiology Department, Hospital de la Santa Creu i Sant Pau, Universitat Autònoma de Barcelona, Carrer St. Antoni M. Claret 167, Barcelona 08025, Spain; Centro de Investigación Biomédica en Red en Enfermedades Cardiovasculares, Av. Monforte de Lemos 3-5, Madrid 28029, Spain

**Keywords:** Aortic dissection, Extracorporeal membrane oxygenation, ECMO, Computed tomography, Case report

## Abstract

**Background:**

Mechanical complications after acute myocardial infarction are associated with high mortality. When a post-infarction ventricular septal defect is complicated by cardiogenic shock and acute aortic dissection during escalation of mechanical circulatory support, multimodality imaging becomes critical for diagnosis and management.

**Case summary:**

A 56-year-old man presented with inferior ST-segment elevation myocardial infarction and underwent urgent coronary angiography, which showed proximal right coronary artery occlusion with only partial reperfusion. Transthoracic echocardiography identified a large inferior ventricular septal defect with a holosystolic left-to-right shunt. Because of haemodynamic deterioration and high surgical bleeding risk after dual antiplatelet therapy, an intra-aortic balloon pump was inserted, followed by femoro-femoral extracorporeal membrane oxygenation for refractory cardiogenic shock. Computed tomography demonstrated type B aortic dissection with partial false lumen thrombosis and showed that the intra-aortic balloon pump had been inadvertently positioned within the false lumen, prompting immediate removal. Computed tomography also revealed extracorporeal membrane oxygenation-related enhancement artefacts. Follow-up computed tomography showed no clear progression. On Day 5, the patient developed refractory ventricular fibrillation. Transoesophageal echocardiography demonstrated severe aortic root and proximal ascending aortic thrombosis, absent aortic valve opening, and minimal residual perfusion. The patient died shortly afterwards.

**Discussion:**

This case highlights the diagnostic and management value of advanced computed tomography in a patient with post-infarction ventricular septal defect, cardiogenic shock, and iatrogenic type B aortic dissection during extracorporeal membrane oxygenation support. It also illustrates important computed tomography pitfalls related to competing blood streams and watershed artefacts under peripheral extracorporeal membrane oxygenation.

Learning points

- Computed tomography during peripheral veno-arterial extracorporeal membrane oxygenation requires awareness of contrast-flow artefacts that may simulate vascular complications.
- Multimodality imaging is essential in post-infarction ventricular septal defect to guide treatment and identify critical complications.

## Introduction

Mechanical complications after acute myocardial infarction are uncommon but carry very high mortality. Ventricular septal defect (VSD) is one of the most catastrophic presentations and frequently leads to cardiogenic shock, often requiring temporary mechanical circulatory support as a bridge to surgery. When acute aortic dissection (AD) develops during escalation of support, management becomes particularly challenging. In this setting, multimodality imaging is essential not only to establish the diagnosis but also to guide management and avoid misinterpretation of support-related findings.

Veno-arterial extracorporeal membrane oxygenation (VA-ECMO) is conventionally considered contraindicated in AD because of the theoretical risk of false lumen (FL) perfusion, dissection propagation, and coronary malperfusion. However, evidence is limited in patients with otherwise unsurvivable cardiogenic shock. This case is of particular educational value because advanced computed tomography (CT), integrated with echocardiography, identified the dissection, demonstrated inadvertent intra-aortic balloon pump (IABP) positioning within the FL, and revealed VA-ECMO-related flow artefacts that could have led to diagnostic error.

## Summary figure

**Figure:**
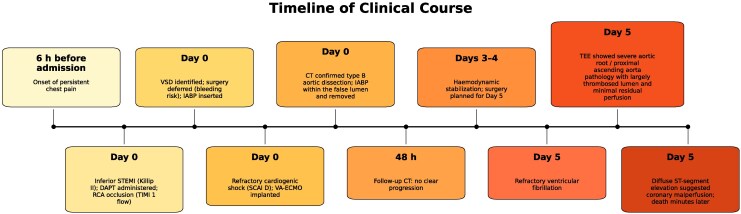


## Case presentation

A 56-year-old man presented to the emergency department with 6 h of chest pain. The patient had a smoking history of more than 20 cigarettes per day and no other known comorbidities. Physical examination showed bibasal crackles, blood pressure of 97/49 mmHg, heart rate of 100 b.p.m., and oxygen saturation of 94%. Electrocardiogram (ECG) demonstrated ST-segment elevation in leads II, III, and augmented Vector Foot. Inferior ST-segment elevation myocardial infarction with Killip class II was diagnosed, and dual antiplatelet therapy (DAPT) was administered before urgent transfer to the catheterization laboratory.

Coronary angiography showed proximal occlusion of the right coronary artery. Despite multiple attempts to cross the lesion, only Thrombolysis in Myocardial Infarction 1 flow was achieved. The patient was then admitted to the intensive cardiac care unit. Immediately after admission, rapid clinical deterioration occurred, and a new Grade 4 holosystolic murmur appeared at the lower left sternal border. Transthoracic echocardiography (TTE) identified a large inferior VSD with a holosystolic left-to-right shunt in parasternal short-axis, modified apical four-chamber, and modified apical two-chamber views (*Figure 1A–1C*; Supplementary material online, *Video S1*). Left ventricular ejection fraction was 46%, and the right heart was dilated with mild right ventricular dysfunction. Immediately after VSD diagnosis, DAPT was suspended because of the anticipated need for surgery and the perceived bleeding risk, and an IABP was inserted. The procedure was technically difficult.

**Figure 1:**
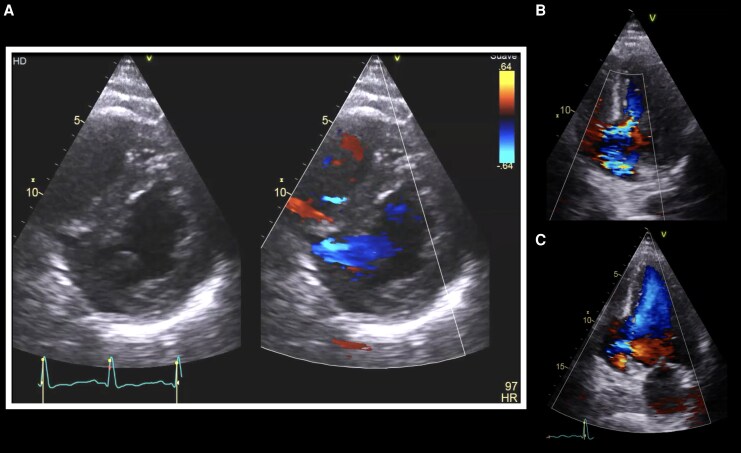
Transthoracic echocardiographic images showing an inferior ventricular septal defect. Colour Doppler flow shows a holosystolic left-to-right shunt in parasternal short axis view (*A*), modified apical four chambers view (*B*) and modified apical two-chamber view (*C*).

Approximately 2 h later, he further deteriorated to refractory cardiogenic shock, requiring orotracheal intubation, invasive mechanical ventilation, and escalating vasoactive support, consistent with Society for Cardiovascular Angiography and Interventions shock stage D. Femoro-femoral VA-ECMO was therefore implanted under transesophageal echocardiogram (TEE) guidance; during this procedure, AD was documented, and urgent CT was performed.

CT proved crucial in defining the anatomy. It demonstrated type B AD according to the Society for Vascular Surgery/Society of Thoracic Surgeons classification,^1^ with the entry tear in Segment 2 and partial thrombosis of the FL (*Figure 2A* and *B*). Importantly, CT also showed that the IABP catheter tip had been inadvertently placed within the FL (*Figure 2C* and *D*), prompting immediate removal. CT also showed the VSD (*Figure 3C*, Supplementary material online, *Video S2*).

**Figure 2:**
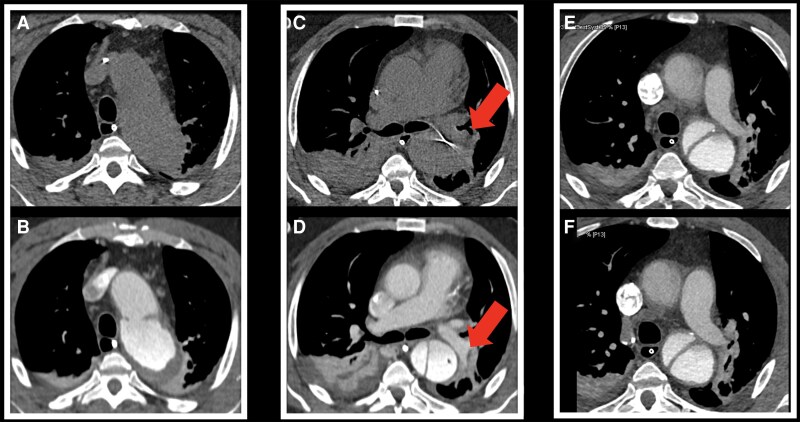
Computed tomography (CT) images showing an aortic dissection and the intra-aortic balloon pump (IABP) catheter placed in the false lumen (FL). The entry and partial thrombosis of the false lumen is detected (*A*, *B*). IABP catheter tip placed in the FL (arrow, *C*, *D*). The outer wall calcification (usually only present in the true lumen) can help to distinguish TL and the FL (*E*, *F*).

**Figure 3:**
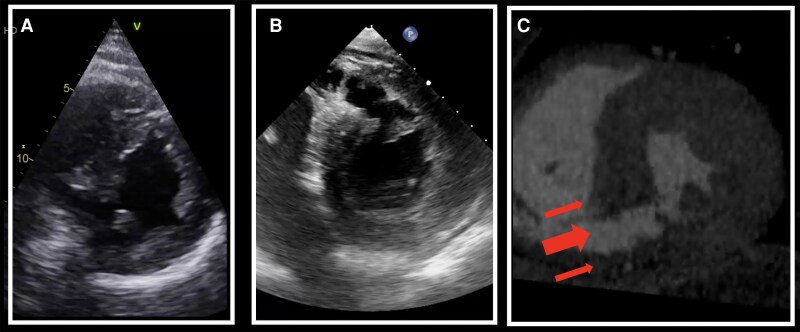
Ventricular septal defect (VSD) compared between different imaging techniques. Transthoracic echocardiogram (TTE) (*A*), transesophageal echocardiogram (TEE) (*B*) and computed tomography (CT) (*C*). Arrows pointing to VSD. Notice hypodense myocardial region (smaller arrows) that also reveal hypoperfused segments.

Alternative strategies could have included earlier surgical intervention despite bleeding risk, device repositioning or removal once malposition was For image description, please refer to the figure legend and surrounding text.identified, or consideration of different support configurations; however, each option carried major risks in the setting of recent DAPT, post-infarction VSD, refractory shock, and acute AD.

CT interpretation was particularly challenging because peripheral femoro-femoral VA-ECMO created two competing blood streams, altering the expected contrast enhancement pattern. This made the distinction between true lumen (TL) and FL difficult on first-pass acquisition. In this context, outer wall calcification, present usually only in the TL, became a key landmark for lumen identification (*Figure 2E* and *F*). CT also demonstrated a watershed artefact caused by the meeting point of the native and extracorporeal circulations, which could have been misinterpreted as pseudolesions such as thrombus or an additional dissection plane (*Figure 4B* and *D*, green arrow). Correlation with non-contrast images and recognition of the true dissection flap were therefore essential (*Figure 4A–4D*, blue arrow).

**Figure 4:**
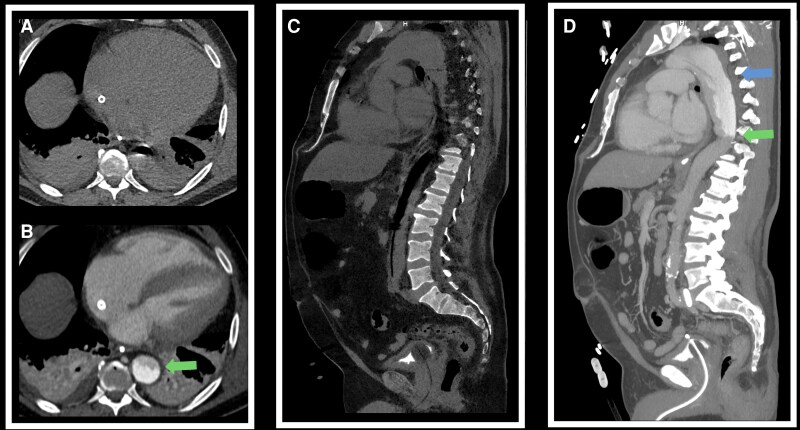
Computed tomography (CT) images showing ‘watershed artefact’ and intra-aortic balloon pump (IABP) catheter in the false lumen (FL). Non-contrast images (diastolic phase, swollen balloon) (*A*, *C*). Contrast images (systolic phase, deflated balloon) (*B*, *D*). The point where the two bloodstreams meet (watershed area, green arrow) can be mistaken for pseudolesions such as thrombus or dissection. The blue arrow shows the real dissection flap.

After IABP removal, the patient remained temporarily stable under extracorporeal membrane oxygenation support, and the aortic valve continued to open. Follow-up CT at 48 h showed no clear progression of the dissection, with persistent patency of both lumens. Vasoactive drugs were progressively reduced, and surgery was scheduled for Day 5.

Before surgery could be performed, the patient developed refractory ventricular fibrillation despite repeated electrical cardioversion. Emergent TEE demonstrated severe pathology involving the aortic root and proximal ascending aorta, with a largely thrombosed lumen, no aortic valve opening, and minimal residual perfusion (*Figure 5A*; Supplementary material online, *Video S3*). In the descending thoracic aorta, abundant spontaneous echocontrast was observed in both lumens (*Figure 5B*; Supplementary material online, *Video S3*), consistent with marked blood stasis. These findings raised concern for either retrograde extension of the dissection into the ascending aorta and aortic root with secondary coronary malperfusion or extensive thrombosis in the setting of severely impaired antegrade flow. Although the final mechanism cannot be established with certainty, the diffuse ST-segment elevation supported acute coronary compromise as the immediate cause of terminal deterioration. In view of the extremely poor prognosis, surgery was not pursued, and the patient died minutes later.

**Figure 5:**
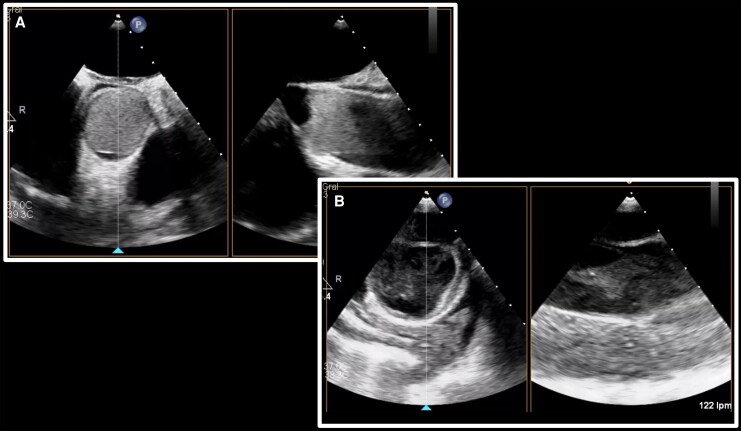
Transoesophageal echocardiography images showing aortic root thrombosis (*A*) and descending thoracic aortic dissection (AD) with abundant spontaneous echocontrast of both true lumen (TL) and false lumen (FL) (*B*).

## Discussion

From a clinical perspective, the case also underscores the dilemma of using VA-ECMO in the presence of AD. Although this is generally considered a contraindication because retrograde flow may worsen FL perfusion or promote extension of the dissection, extracorporeal membrane oxygenation was instituted here in a setting of otherwise unsurvivable cardiogenic shock.^2^ However, the terminal event demonstrated the potential for catastrophic aortic root complications and probable coronary malperfusion.

This case illustrates the value of multimodality imaging in a patient with post-infarction VSD, refractory cardiogenic shock, and iatrogenic type B AD during escalation of mechanical circulatory support. Its main educational message lies in the imaging pathway: echocardiography established the initial mechanical complication, whereas advanced CT defined the aortic injury, identified device malposition, and revealed extracorporeal membrane oxygenation-related artefacts with major diagnostic implications. The case also adds to prior reports showing the importance of multimodality imaging in complex post-myocardial infarction mechanical complications.^3^

First, TTE rapidly confirmed the diagnosis of post-infarction VSD by demonstrating a large inferior defect with a holosystolic left-to-right shunt. Second, CT was decisive once an aortic complication was suspected. Beyond confirming type B AD, it identified the entry tear, documented partial FL thrombosis, and, most importantly, showed the IABP catheter within the FL. This finding directly changed management because the device was removed immediately.

Third, this case highlights the specific interpretative challenges of CT in patients on peripheral VA-ECMO. The coexistence of native cardiac output and retrograde extracorporeal flow produces two blood streams with different contrast opacification patterns. Their meeting point, the watershed area, may generate first-pass artefacts that mimic thrombus, vascular occlusion, or additional dissection planes.^4^ Classic echocardiographic indicators, such as a smaller area of the TL compared to the FL or systolic expansion of the TL, may be altered in this context. Likewise, discrimination between true and FL may be more difficult than in standard dissection imaging.^5^ In our patient, recognition of the true dissection flap and use of outer wall calcification as a marker of the TL were essential to avoid misinterpretation. In some cases, other CT signs supporting FL identification could be partial thrombosis and the cobweb sign, defined as thin residual linear structures within the FL. Careful comparison with non-contrast images, delayed-phase acquisitions when available, and multi-planar assessment of the real intimal flap can help distinguish flow-related artefact from true pathology.^6^

Overall, this report is particularly relevant for an advanced CT audience because it emphasizes that CT interpretation under VA-ECMO requires understanding of extracorporeal-flow haemodynamics, integration with non-contrast acquisitions, and correlation with echocardiography. Failure to recognize these features may lead to diagnostic error in critically ill patients.

## Supplementary Material

ytag524_Supplementary_Data

## Data Availability

The data underlying this article are available in the article and in its online Supplementary material.

## Lead author biography

Dr Adrià Ruiz-López graduated in 2017 from the Faculty of Medicine of the Autonomous University of Barcelona, Spain. He is currently a cardiologist at Hospital de la Santa Creu i Sant Pau and Hospital Universitari Mútua de Terrassa in Barcelona, Spain. His main areas of interest are advanced cardiac imaging, cardiomyopathies, and sports cardiology.

## Notes

**Consent:** Written informed consent was obtained from the patient’s next of kin for publication of this case report and any accompanying images, in accordance with COPE guidelines.
