# Supplementary material for: Computed tomography pitfalls and diagnostic value in a patient with post-infarction ventricular septal defect, cardiogenic shock, and iatrogenic type B aortic dissection during extracorporeal membrane oxygenation support: a case report
Source: Eur Heart J Case Rep. 2026 Jul 14;10(7):ytag524. doi: 10.1093/ehjcr/ytag524 (PMC13422630; doi:10.1093/ehjcr/ytag524)
Supplement: ytag524_Supplementary_Data [file ytag524_supplementary_data.zip › Video captions.docx]

**Alternative text (supplementary material)**

**Video 1. Transthoracic echocardiographic images showing an inferior ventricle septal defect.** Color doppler flow shows an holosystolic left-to-right shunt in parasternal short axis view **(A)**, modified apical four chambers view **(B)** and modified apical 2 chambers view **(C)**.

**Video 2.** VSD compared between different imaging techniques. ETT **(A),** TEE **(B)** and CT **(C)**.

**Video 3. Transesophageal echocardiography images showing aortic root thrombosis (A) and descending thoracic AD with abundant spontaneous echocontrast of both TL and FL (B).** Notice no aortic valve opening is present.
